# Impediments and Discretion During Management Perplexities in Cases of Dental Impactions: A Case Series

**DOI:** 10.7759/cureus.68756

**Published:** 2024-09-05

**Authors:** Deval K Arora, Jay K Somkuwar, Tarang Chadha, Pallavi Parasramka

**Affiliations:** 1 Dentistry, Autonomous State Medical College, Shahjahanpur, IND; 2 Pedodontics and Preventive Dentistry, Sardar Patel Post Graduate Institute of Dental and Medical Sciences, Lucknow, IND; 3 Pedodontics and Preventive Dentistry, King George's Medical University, Lucknow, IND

**Keywords:** bicuspid, dental anomalies, impacted, maxilla, supernumerary

## Abstract

Impacted teeth are significant dental anomalies affecting primary and mixed dentition, potentially causing issues with permanent teeth and affecting dental and facial aesthetics. Supernumerary teeth are commonly impacted in the anterior maxilla, while premolars are the third most common in permanent dentition, after third molars and canines. Untreated impactions can lead to complications such as delayed or ectopic eruption of adjacent teeth, resulting in crowding, midline diastema, or nasal cavity eruption. Early diagnosis and prompt treatment are essential to prevent these issues. Despite advanced surgical techniques, some complications can be iatrogenic, making awareness and preventive measures crucial. This article presents a series of cases highlighting common complications and precautions associated with the management of dental impactions. Timely diagnosis and proactive management can mitigate complications from impacted teeth. Dental surgeons should remain vigilant for clinical signs and unexpected findings during routine examinations.

## Introduction

Impaction occurs when teeth fail to erupt into the dental arch within the expected time due to factors like the position and size of adjacent teeth, dense overlying bone, excessive soft tissue, or genetic abnormalities [1]. Supernumerary teeth, which are extra teeth beyond the normal set, most commonly impact the anterior maxilla, with a prevalence of 0.3-0.8% in primary dentition and 0.1-3.8% in permanent dentition [2]. About 90% of these teeth are found in the maxilla, while only 10% are in the mandible [3]. Males are more frequently affected (62.2-87%) than females (13%-37.8%) [3]. According to the dichotomy theory, Taylor proposed that a tooth bud can split into either two equal-sized teeth or one normal and one dysmorphic tooth [2]. Another well-supported theory, the hyperactivity theory, posits that supernumerary teeth result from local, independent conditioned hyperactivity of the dental lamina. Specifically, a lingual extension of an additional tooth bud results in a eumorphic tooth, while a rudimentary form arises from the proliferation of epithelial remnants of the dental lamina, triggered by the pressure of the complete dentition [2].

Supernumerary teeth are commonly associated with conditions such as cleft lip and palate, cleidocranial dysplasia, Gardner’s syndrome, and Ellis-van Creveld syndrome [3]. Four morphological types of supernumerary teeth are described in the literature [2,3]: conical (peg-shaped, mesiodens); tuberculate (barrel-shaped, with tubercles on the crown); supplemental (duplication of teeth in the normal series); and odontoma (odontogenic tumor, hamartomatous malformation).

In addition to supernumerary teeth often showing ectopic eruptions and impactions, permanent teeth impactions can also affect any tooth necessitating extraction. The most frequently impacted teeth are the mandibular and maxillary third molars, followed by the maxillary canines and premolars. Impacted premolars in adults have a prevalence of 0.5% (0.1-0.3% for maxillary premolars and 0.2-0.3% for mandibular premolars) [4]. Premolar impactions may result from factors such as mesial drift due to premature loss of primary molars, ectopic positioning of tooth buds, or pathologies like inflammatory or dentigerous cysts [4].

Untreated impacted teeth usually follow complications including delayed eruption, ectopic eruption of adjacent teeth, midline diastema, malalignment of incisors, displacement, axial rotation of adjacent teeth, radicular resorption, crowding, dilacerations, development of dentigerous cysts, and migration into the nasal cavity or maxillary sinus [5,6].

Although many impacted teeth can be removed with instruments like elevators, forceps, etc., potential complications should not be overlooked, as they can cause significant distress to patients and surgeons. Factors such as the impacted tooth's position and its relationship to adjacent teeth, the maxillary sinus, blood vessels, nerves, and anatomical spaces play crucial roles in complication development [6]. Despite surgical expertise, some complications may be iatrogenic, so awareness of potential issues can aid in their prevention.

The present manuscript reports two cases of multiple supernumerary impacted teeth in male children and one case of an impacted maxillary second premolar in a male child, presenting a total of three cases (case series) depicting management conundrums during the treatment of dental impactions. It includes the successful management of these cases and discusses potential complications and the precautions to be taken during and post-surgery of impacted teeth.

## Case presentation

Case 1

**Figure 1:**
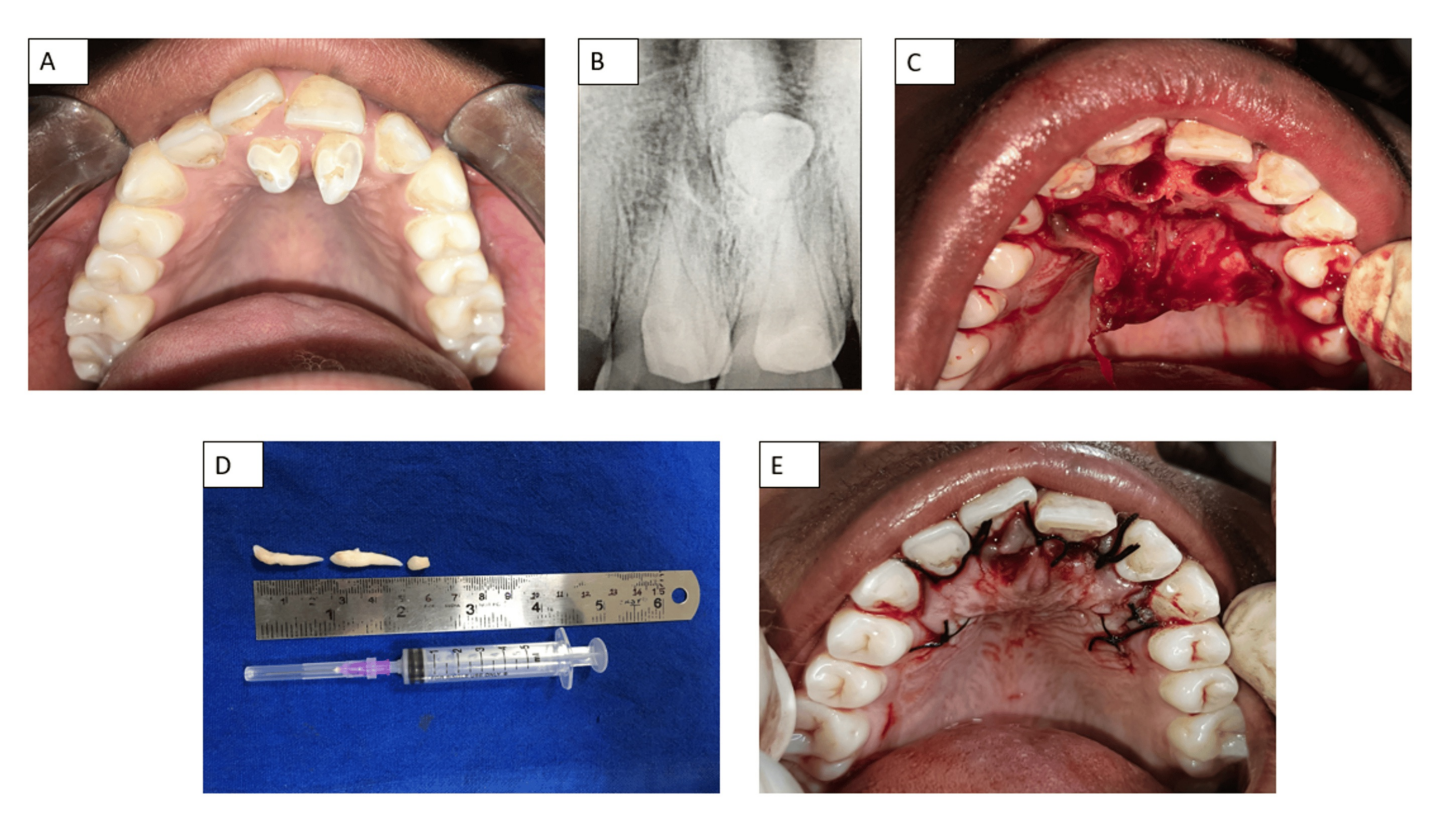
(A) Preoperative view of maxillary anterior supernumerary teeth (tuberculate type); (B) Intra-oral periapical radiograph showing three supernumeraries; (C) Palatal full-thickness mucoperiosteal flap raised; (D) Extracted three supernumerary teeth; (E) Postoperative suturing with 3-0 silk sutures

A 15-year-old boy presented to the Department of Dentistry with a primary complaint of difficulty in chewing due to extra teeth in the upper front region of his jaw. Medical, dental, and family histories were unremarkable. Intraoral examination revealed two supernumerary teeth (tuberculate type) on the palatal aspect of the maxillary central incisors (Figure 1A). Further radiographic examination showed an inverted, embedded, conical-shaped supernumerary tooth (Figure 1B).

After obtaining written and informed consent from the guardians and verbal assent from the child, transalveolar extractions were planned to remove all three supernumerary teeth. Complete blood count (CBC) investigation reports were obtained with a focus on the normalcy of hemoglobin levels and normalized bleeding and clotting times (BT/CT) along with a sensitivity check for local anesthesia allergy by performing an intradermal local anesthetic (LA) injection test. Initially, the two erupted supernumeraries were extracted under bilateral infraorbital nerve block and nasopalatine nerve block. A palatal full-thickness mucoperiosteal flap was raised with two vertical releasing incisions in the region of the canines bilaterally. Respective crevicular incisions were then made, and a soft tissue flap was elevated to access the third inverted impacted supernumerary tooth (Figure 1C). An osteotomy was performed with copious saline irrigation to facilitate access to the palatally embedded tooth, followed by luxation and removal (Figure 1D). Bleeding control was done with pressure packs. The palatal flap was repositioned and sutured with 3-0 silk sutures (Figure 1E). Postoperative antibiotics and analgesics along with regular warm saline rinses, a convalescent diet, and thorough oral hygiene practices were prescribed, with a detailed live demonstration of the modified Bass method of toothbrushing, and the use of an ultrasoft bristled toothbrush for hygiene maintenance was recommended. The patient was scheduled for suture removal after seven days, which was followed by decent soft tissue healing during the recall session due to meticulous patient compliance.

Case 2

**Figure 2:**
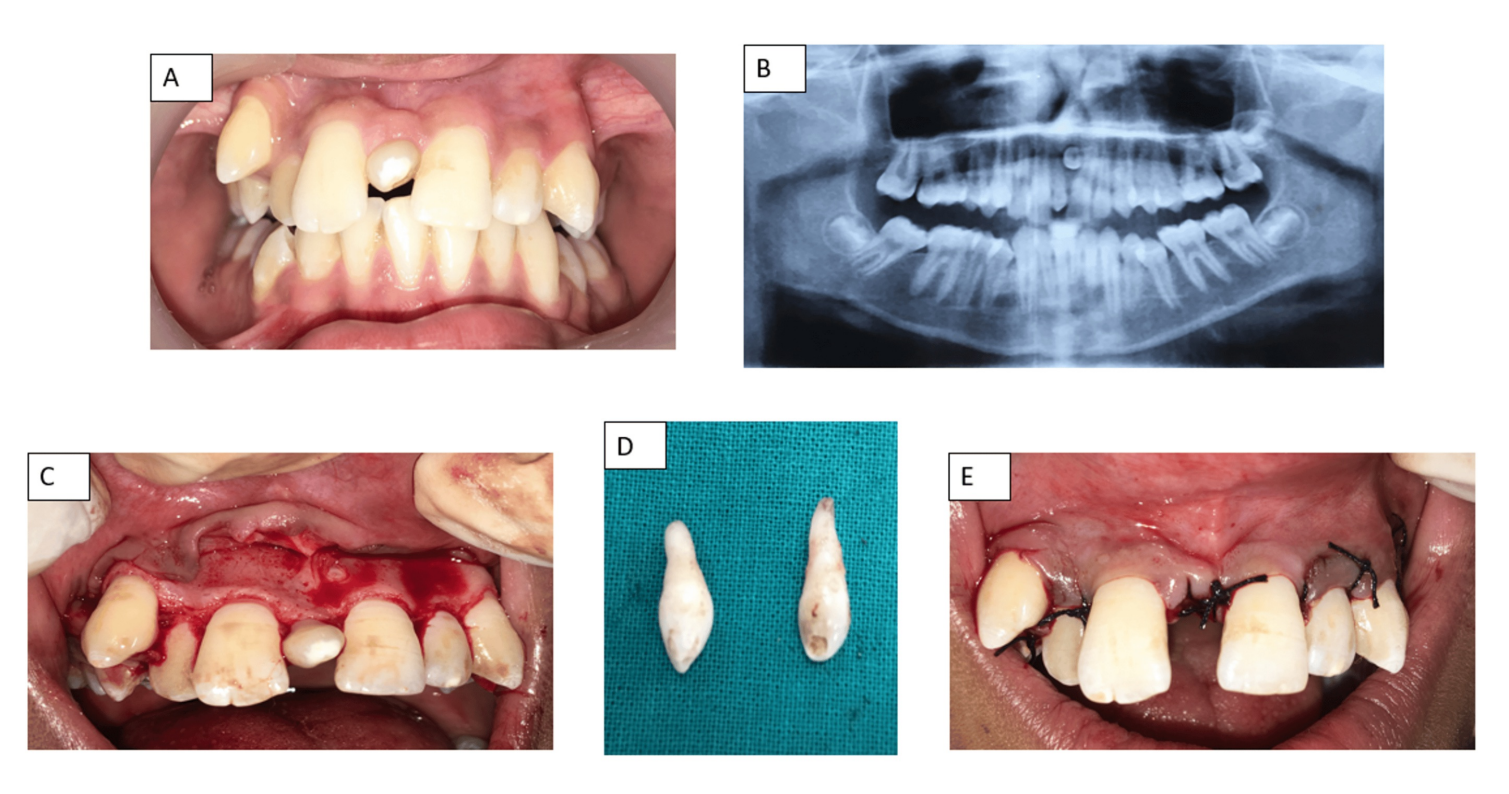
(A) Preoperative view of the maxillary anterior supernumerary tooth (conical type); (B) Orthopantomogram showing two supernumeraries; (C) Labial full-thickness mucoperiosteal flap raised; (D) Extracted two supernumerary teeth; (E) Postoperative suturing with 3-0 silk sutures

A 13-year-old boy presented to the Department of Dentistry with a primary concern of maligned upper front teeth. The patient's medical, dental, and family histories were unremarkable. Intraoral examination revealed mesiodens (conical) situated between the maxillary central incisors (Figure 2A). An orthopantomogram was used to identify an inverted, conical-shaped impacted supernumerary tooth located between the apices of the erupted mesiodens and the left central incisor (Figure 2B). After obtaining written and informed consent from the guardians and verbal assent from the child, surgical extractions were planned to remove both erupted and impacted supernumerary teeth, respectively.

CBC, hemoglobin, and BT/CT levels were found to be within normal parameters. Additionally, an intradermal LA injection test was done to check for allergic sensitivity.

A full-thickness labial mucoperiosteal flap was elevated from the distal aspect of the canine on one side to the distal aspect of the canine on the other side, with respective vertical and crevicular incisions to facilitate visualization of the impacted supernumerary tooth (Figure 2C). First, the erupted mesiodens was extracted post a bilateral infraorbital and nasopalatine nerve block, followed by the bone guttering with saline irrigation to gain access to luxate and remove the inverted impacted supernumerary tooth from within the bone (Figure 2D). Hemorrhagic balance was achieved by pressure packs. The labial flap was then repositioned and sutured with 3-0 silk sutures (Figure 2E). Postoperative antibiotics and analgesics with lukewarm saline rinses to enhance healing and hygiene, a soft diet, and careful oral sanitation practices were prescribed, with an elucidation of the modified Bass method of tooth cleaning, and utilization of an ultrasoft bristled toothbrush for oral hygiene was suggested. The patient was scheduled for suture removal after a week. The following appointment showed adequate healing post suture removal.

Case 3

**Figure 3:**
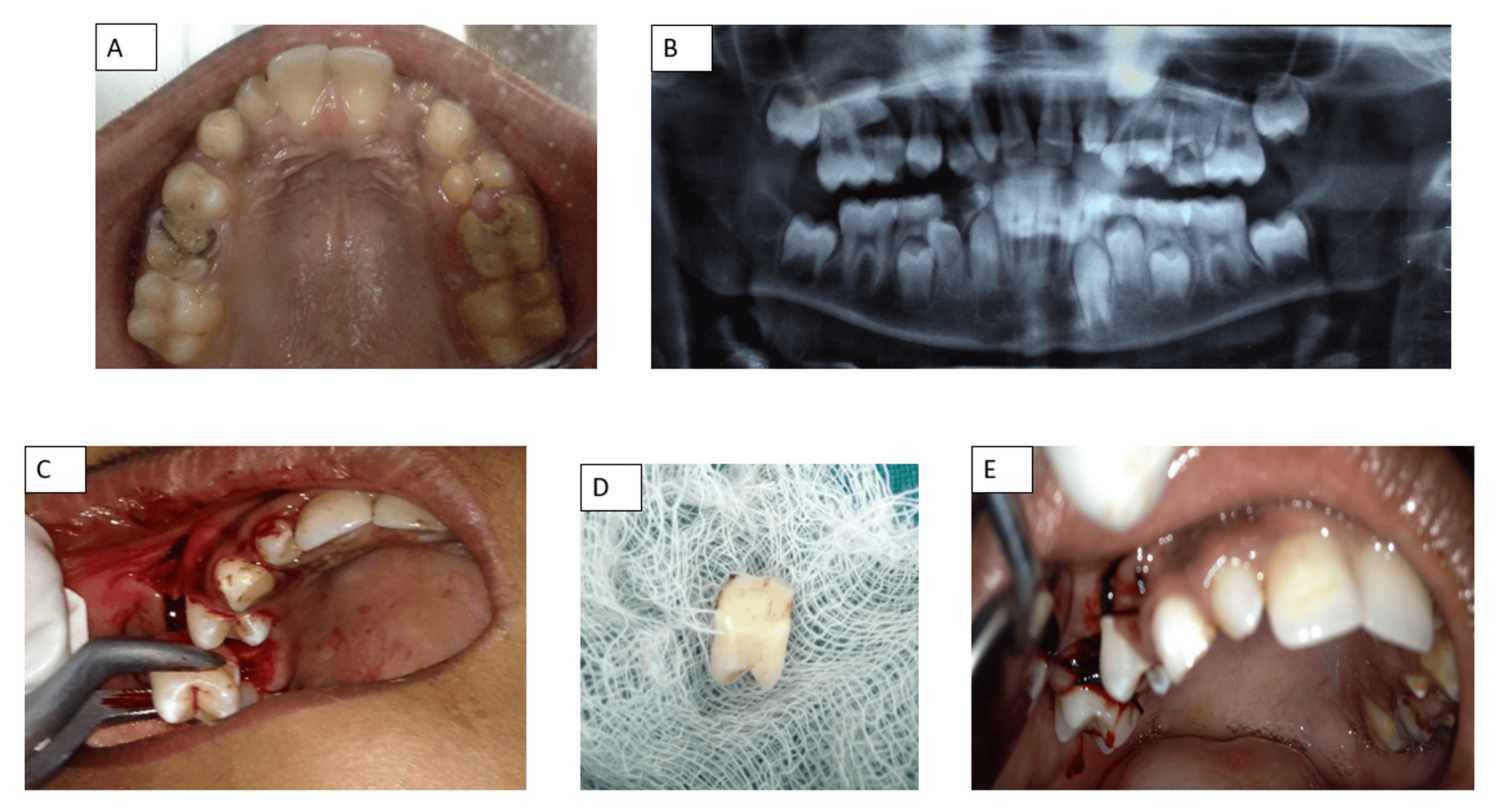
A to E: (A) Preoperative view showing carious 55 and 65; (B) Orthopantomogram showing impacted 15 with unusual transverse long axis and incompletely formed root; (C) Buccal full-thickness mucoperiosteal flap raised; (D) Extracted maxillary right second premolar; (E) Postoperative suturing with 3-0 silk sutures

A 12-year-old boy presented to the Department of Dentistry with a chief complaint of pain in the right upper back tooth region of the jaw. The past medical, dental, and family histories were unremarkable. Intraoral examination revealed a grossly decayed and retained maxillary right primary second molar (tooth 55) and carious, retained primary mandibular right first molar (tooth 84), as well as primary maxillary left second molar (tooth 65) (Figures 3A-3B). An intraoral periapical radiograph showed severe destruction of tooth 55, including involvement of the furcation area. Additionally, a horizontally inclined impacted maxillary right second premolar (tooth 15) with an unusual transverse long axis and incompletely formed root was also observed. A panoramic radiograph (orthopantomogram) was taken to confirm these findings and to determine the exact location of the impacted maxillary second premolar. Following written and informed consent from the guardian and assent from the child, a transalveolar extraction was planned for the removal of the impacted tooth 15 along with grossly carious tooth 55 since pulpectomy was not an option.

CBC, hemoglobin, and BT/CT reports were found to be within the normal range. Pre-surgical intradermal LA injection testing was done to be certain of any allergic sensitivity.

To begin, tooth 55 was extracted after administering an infraorbital nerve block and a greater palatine nerve block. A buccal full-thickness mucoperiosteal flap was then meticulously raised, extending from the mesial papilla of the first premolar to the distal papilla of the first permanent molar on the same side employing adequate vertical releasing and crevicular incisions with periosteal elevators (Figure 3C). Bone osteotomy with irrigant as saline was done on the buccal aspect of the maxillary bone to gain visual access to the impacted maxillary right second premolar. The tooth was adequately elevated and surgically extracted (Figure 3D). Hemorrhage was controlled with pressure packs. The buccal flap was repositioned and sutured with 3-0 silk sutures (Figure 3E). Postoperative antibiotics and analgesics with warm saline rinses to accelerate healing, a nutritionally rich diet, and a guarded oral sanitation routine were prescribed, along with a depiction of the modified Bass method of tooth hygiene, and the use of an ultrasoft bristled tooth cleaning aid to enhance oral disinfection was recommended. The patient was scheduled for a follow-up visit in seven days for suture removal. Definitive healing of soft tissues was observed post suture removal at the consecutive visit of the patient giving credence that the patient had followed all postoperative instructions with care.

## Discussion

Complications related to the removal of impacted teeth are significant and influenced by various factors, including tooth position, patient age and health, surgeon experience, and equipment quality. These complications can be categorized into those occurring during surgery and those arising afterward. 

Complications during impacted tooth extractions

Tooth and Adjacent Tooth Issues

Tooth fractures: Impacted or adjacent teeth may fracture despite careful use of elevators or forceps. The variability in root shape can lead to fractures of the impacted tooth or its roots. Displacement of adjacent teeth is common and often results from excessive force or loss of supporting bone. Displaced teeth should be repositioned and immobilized for 3-4 weeks, with a soft diet recommended. Deeply impacted mesiodens may also be dispatched into the nasal cavity or maxillary sinus due to inadvertent forces, wherein endoscopic techniques are the golden standard for retrieval of the same in the present times [1,7].

Soft Tissue Complications

Injuries and hemorrhage: Injuries to soft tissues, bleeding, and hematoma formation are possible. Hemorrhage can be local or systemic, influenced by tooth position, inclination, and patient age. Older patients and deeply impacted teeth are at higher risk [7]. Hematomas vary in size and origin and may require regular follow-up. These complications can be avoided by using proper aspiration technique while injecting local anesthesia and evading the use of unnecessary excessive force during luxation [7]. Surgical emphysema, caused by high-speed turbines forcing air into soft tissues, clinically depicted as swelling and crepitation should be managed with low-speed handpieces and sterile saline irrigation during osteotomy and tooth separation [8].

Nerve Injuries

Types of nerve damage: Nerves may suffer from traumatic, compressive, or toxic injuries, leading to conditions like neuropraxia, axonotmesis, or neurotmesis. Neuropraxia involves transient impairment of the myelin sheath, while axonotmesis and neurotmesis involve more severe nerve damage. Symptoms may include numbness, paraesthesia, dysesthesia, or hypoesthesia [9]. The causes include direct trauma to the nerve from the needle, intraneural hematoma, or local anesthetic toxicity. Air blasts during the cleaning of an extraction site should also be avoided [10].

Maxillary Sinus Complications

Sinus issues: Extraction of impacted maxillary teeth can lead to maxillary sinusitis or oroantral fistula formation if an oroantral communication is present. Management may involve specialized procedures such as buccal sliding mucoperiosteal flaps, bone grafts, or synthetic materials. Patients should follow postoperative care instructions, including avoiding nose blowing and smoking [1,11].

Surgical Equipment Issues

Instrument failures: Equipment complications often result from metal fracturing due to heat or torsion. Excessive force during surgery can also cause instrument breaks. Fractured instruments should be promptly removed [1].

Swallowing and Aspiration

Tooth fragments: Swallowing or aspiration of tooth fragments is rare, with an incidence of about 0.004% [11]. Swallowed objects usually pass through the gastrointestinal tract without issues, but if symptoms like pain, vomiting, or tenderness occur, surgical intervention may be necessary. Aspiration is less common but may require immediate referral to a pulmonologist and chest X-rays to diagnose and manage airway obstruction [11].

Complications following impacted tooth extraction

Post-surgery, common postoperative sequelae include pain, swelling, trismus, hemorrhage, and dry socket. Risk factors like age, tooth position, and procedure duration influence morbidity, while female patients may face additional challenges due to anatomical and hormonal factors. Assistant handling can also impact outcomes by affecting normal lymph drainage if the cheek or soft tissue retractors are manipulated with brute force resulting in unnecessary swelling [12]. Postoperative infections and inflammatory conditions, such as abscesses, alveolar osteitis, and osteomyelitis, occur in 1-30% of cases [12]. Bacteria-related fibrinolysis can prevent blood clot formation resulting in dry sockets; however, chlorhexidine rinses pre- and post-surgery have been shown to reduce dry socket incidence [12,13].

Impacted supernumerary teeth overview

Supernumerary teeth, like mesiodens, are rare without systemic conditions [5]. Our series of cases found no syndromes, with affected patients being predominantly male, which was in accordance with Anthonappa et al. [14]. Treatment depends on the child's age, clinical presentation, and root development of adjacent teeth [15]. Asymptomatic mesiodens are often left until adjacent teeth's roots are formed to avoid iatrogenic damage to permanent teeth, which may result in loss of vitality, arrested root development, loss of lamina dura, and bone deformities [16]. In the present article also, the impacted supernumerary teeth were extracted after complete root formation of maxillary central incisors. However, according to Rajab and Hamdan [2], extracting mesiodens early improves prognosis and reduces risks of complications like delayed eruption, root resorption, midline diastema, and bone deformities.

Extraction approaches

For highly inverted impactions, the labial approach offers better visibility and less surgical time, though it may cause aesthetic issues (due to gingival recession) and increased swelling [17,18]. The palatal approach reduces aesthetic concerns, and the postoperative swelling is less due to the presence of dense soft tissue on the palatal side but can lead to more intraoperative bleeding due to the presence of palatine vasculature, leading to prolonged surgery [19]. In the current manuscript, case 1 was done using the palatal approach technique, resulting in less swelling but increased bleeding, compared to the labial approach technique in case 2. In all three instances, the neighboring teeth showed positive responses to electric pulp testing, which was used to evaluate the postoperative vitality of the adjacent teeth. Additionally, thorough surgical planning with avoidance of excessive bone cutting and use of force (minimally invasive technique), enabled rapid bone healing, facilitating a seamless transition to later orthodontic treatment, furthermore curbing malalignments.

## Conclusions

Effective management of impacted teeth is crucial to prevent complications such as delayed eruption, malalignment, and damage to adjacent structures. Early diagnosis and timely intervention are essential in mitigating risks and achieving favorable outcomes. Surgical techniques should be chosen based on the specific characteristics of the impaction and the anatomical considerations to minimize complications. Despite advancements in surgical approaches, vigilance during procedures and thorough postoperative care are critical to address and prevent potential complications. Dental surgeons must remain attentive to the signs of potential issues and adapt their strategies to individual patient needs for optimal results.
